# Are Babies Born Preterm High-Risk Asthma Candidates?

**DOI:** 10.3390/jcm12165400

**Published:** 2023-08-19

**Authors:** Carlo Caffarelli, Serena Gracci, Giuliana Giannì, Roberto Bernardini

**Affiliations:** 1 Clinica Pediatrica, Azienda Ospedaliero-Universitaria, Department of Medicine and Surgery, University of Parma, 43126 Parma, Italy; 2 Pediatric Unit, San Giuseppe Hospital, 50053 Empoli, Italy

**Keywords:** asthma, preterm, allergy, bronchiolitis, lung function, microbiome, atopy, breastfeeding, smoking

## Abstract

Among preterm infants, the risk of developing asthma is a matter of debate. This review discusses the state of the art of poorly understood prematurity-associated asthma. Impaired pulmonary function is common in children born prematurely. Preterm infants are prone to developing viral respiratory tract infections, bronchiolitis in the first year of life, and recurrent viral wheezing in preschool age. All of these conditions may precede asthma development. We also discuss the role of both atopic sensitization and intestinal microbiome and, consequently, immune maturation. Diet and pollution have been considered to better understand how prematurity could be associated with asthma. Understanding the effect of factors involved in asthma onset may pave the way to improve the prediction of this asthma phenotype.

## 1. Introduction

**Figure 1:**
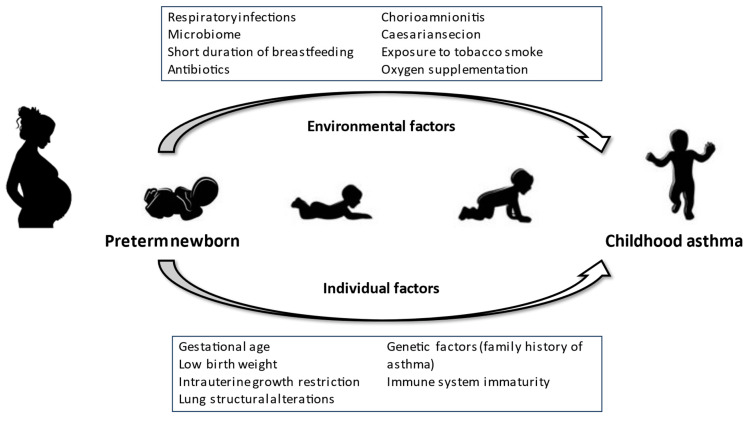
The effects of environmental and individual factors leading the preterm newborn to the development of childhood asthma.

Asthma is a heterogeneous disease, usually characterized by chronic airway inflammation, defined according to the GINA recommendations by the history of respiratory symptoms, such as wheezing, shortness of breath, chest tightness, and cough, which vary over time and in intensity, together with variable expiratory airflow limitation [1]. Asthma is the most common chronic disease in childhood and one of the most frequent chronic diseases in all ages, as it is estimated that 358 million people are affected worldwide, with geographical differences gradually narrowing. The Global Asthma Network (GAN) Phase I study determined a prevalence of current wheeze of 11.1% among adolescents and 9.1% in children and asthma ever of 10.5% and 7.5%, respectively [2]. In children, the most common asthma phenotype is type-2 (T2)-high, characterized by eosinophilic airway inflammation. T2-high comprises the allergic phenotype, about 80% of cases, due to sensitization to allergens that elicit a Th2 response with an IgE production. The T2-high non-allergic phenotype is not IgE-mediated. T2-low is distinguished by a neutrophilic infiltrate that is rare in childhood and commonly begins in adulthood. Preterm infants are born before 37 weeks gestational age (wGA) is completed. They can be classified as extreme (<28 wGA), very (28–32 wGA), or moderate to late (32–36 wGA) preterm [3]. It has been estimated that global preterm delivery is growing especially in industrialized countries [4] from about 9.8% of births in 2000 to 14% in 2014, with 81% occurring in Asia and sub-Saharan Africa [5]. Preterm birth is the main cause of mortality under 5 years of age. Most fatalities occur in the neonatal period [6]. However, the advances in neonatal intensive care management have increased survival rates among premature newborns up to more than 95% [7,8], but they have increased morbidity [9]. Preterm birth can be associated with altered development of the lung and morbidity in the respiratory tract, including respiratory infections, wheeze, and asthma [10,11,12,13]. This is a narrative review performed with a literature search of the MEDLINE database using the following keywords: asthma, preterm delivery, prematurity, and gestational age. We aimed to characterize the occurrence of asthma in preterms and identify preterm infants who are at risk of asthma. We identified many factors associated with the development of asthma in preterms; some of them can be present both in preterm and in term children, sometimes more frequent among the former, while others are exclusive to prematurity. They are both environmental and individual factors that can intervene in various moments of the pre- and postnatal life of the preterm (Figure 1). The extent of the effect of these factors is different from each other; for some of them, it is minimal, while others significantly modify the risk of developing asthma (Table 1).

## 2. Frequency of Asthma in Preterm

The increased susceptibility to asthma in preterm infants has been shown by several studies. Jaakkola, in a systematic review that included 19 studies comparing preterm to term infants, found that preterm infants were 7% more likely to develop asthma according to a fixed-effects model, and the risk reached 36% in a random-effects model [14]. Most studies have focused on the development of asthma at different pediatric ages. In a retrospective cohort study of 7925 infants, late preterm compared to term infants had a 1.7-fold increased risk of persistent asthma at 18 months of age [15]. In the U.S., a large prospective birth cohort study found that preterm birth was associated with a higher prevalence of asthma at ages 0–5 and 6–9 years [16]. An Alaskan population-based study comparing preterm birth <32 wGA to full-term birth revealed that the former was twofold more likely to have asthma at both 0–5 years of age and 6–9 years [17]. These results were confirmed among late preterm infants in a prospective study showing that they were threefold more likely to develop asthma compared to term infants at 7–8 years of age [18] In a retrospective case–control study in 44,173 infants, very preterm infants (<32 wGA) were 3.9-fold more likely to have asthma, and moderate–late preterms (33–36 wGA) were 1.7-fold more likely compared to term infants (39–40 wGA) at 0–19 years of age [19]. A retrospective study on 90,721 children found that preterm infants were 1.64-fold more likely to have asthma compared with term infants at age 0–17 [20]. Furthermore, the increased risk of asthma also persists in adulthood. A Swedish national cohort study, which followed more than 4,000,000 subjects from birth to 46 years of age [21], showed an increased risk of asthma associated with preterm birth in children in each age group (<10, 10–17, 18–46 years).

## 3. Gestational Age

The immaturity represented by the gestational age is a key factor. The risk of asthma increases with decreasing gestational age, as shown by a large birth cohort study, in which children born <31 wGA were 3.2–6.2 times more likely to develop asthma and those born 32–36 wGA were 1.5–2.5 times more likely when compared to term infants [16]. These findings have been confirmed among adolescents (13–14 years of age), in which those born very preterm compared with those born moderate–late preterm required more respiratory admissions, were more likely to have current asthma (21.6% vs. 9.5%, *p* = 0.04), and had significantly reduced lung function compared with those born moderate–late preterm [22]. The relative risk of asthma medication prescriptions, indirectly reflecting the asthma severity, for infants with extreme prematurity was 1.92-fold that of infants with moderate–late preterm status [23]. Low birth weight (LBW) (<2500 g) is an additional risk factor for developing asthma [24,25], and the risk rises with weight reduction. Infants with extremely LBW (<1000 g) have a 1.8-fold increased risk of prescription of asthma medications. Very LBW (<1500 g) infants have a 1.43-fold higher risk than LBW infants [23]. Intrauterine growth restriction (IUGR), often associated with preterm birth, represents an additional independent risk factor for future asthma [26]. Most LBW babies also show catch-up growth in infancy, which was associated with lower lung function and an increased risk of childhood asthma [27,28]. A large meta-analysis showed that this increased risk is not explained by preterm birth alone, but high childhood weight gain (>700 g/month) is an independent risk factor for preschool wheezing and school-age asthma [29]. 

## 4. Airway Impairment and Asthma Development

Many studies have investigated the mechanisms that lead to the development of asthma in preterm children. Prematurity determines an alteration of the lung development, which consists of five phases: the embryonic period (up to week 6), the pseudoglandular period (weeks 6–16), the canalicular period (weeks 16–24), the saccular period (weeks 24–40), and the alveolarization period (mainly after birth) [30]. In preterm infants, the abnormal maturation of the lung at birth especially before the 32nd week [31] leads to lung function impairments. Mechanical ventilation was associated with bronchial hyperresponsiveness in very LBW infants (<1500 g) who had altered alveolar development and hypertrophy of the bronchial smooth muscle [32]. Preterm infants compared to those born at term had lower lung function in childhood, adolescence, and adulthood [32,33,34,35,36]. The Tasmanian Longitudinal Health Study showed that very-to-moderate prematurity was associated with obstructive lung function deficits including chronic obstructive pulmonary disease (COPD) into the sixth decade of life [37]. These alterations in lung function are independent of lung damage at birth and may represent the result of exposures to maternal smoking, oxygen by a ventilator, and treatment with microbiome-altering therapies [38]. In children born at term, an increased neonatal resistance compared to term controls was associated with wheezing until age 3 years of infancy, while reduced neonatal compliance was associated with wheezing and asthma up to 5 years of life [39]. Furthermore, a lung function deficit and increased bronchial responsiveness to methacholine in infants born at term were risk factors for asthma at 7 to 10 years of age [40,41]. Interestingly, low lung function parameters at the age of six months predicted wheezing and hospitalization for wheezing in schoolchildren born preterm [42]. Children born preterm have more respiratory symptoms, including cough and wheezing than controls before adolescence, irrespective of bronchopulmonary dysplasia (BPD), a chronic lung disease that mainly affects extremely preterm infants, which is the most common complication of prematurity, predisposing the infants to the development of obstructive pulmonary diseases [43,44,45,46,47,48,49,50]. Several studies showed an increased prevalence of asthma in children with a history of BPD [23,51,52], and reduced lung function was less evident in children without BPD than in BPD subjects [36]. However, it is controversial whether the asthma-like symptoms of children with BPD can be diagnosed as asthma or whether BPD and asthma should be considered different entities having different pathogenetic pathways [10,53,54]. In children born preterm, asthma should be differentiated from wheezing due to structural changes in lung parenchymal related to prematurity or poor intrauterine lung growth, leading to fixed airflow obstruction [54,55,56], especially when exhaled nitric oxide levels are low and eosinophilic inflammation is lacking [57,58,59,60]. Intrauterine inflammation to which premature infants are exposed [61] and postnatal factors such as hyperoxia and overdistension [62] can interfere with airway development, which will present a reduced caliber. Neonatal oxygen supplementation is a risk factor for asthma in very LBW infants (<1500 g) [32]. These factors might also trigger an inflammatory process that becomes persistent [56,63]. Along this line, children born <32 wGA, compared to full-term children, have increased lower airways neutrophilic inflammation [64], which is found more often in adult asthma and very rarely in childhood asthma and thus is usually eosinophilic, suggesting a different pathway leading to the development of asthma [65]. It is of note that inflammation linked to asthma in obesity needs to be investigated in premature children [66].

## 5. Environmental Agents Associated with the Development of Asthma in Preterm Infants

The increased susceptibility to asthma of preterm infants is determined by numerous factors, which could have important clinical consequences already from the fetal stage and in the first periods of life.

### 5.1. Allergic Sensitization

Allergic sensitization to aeroallergens may occur less frequently in preterm infants. Siltanen et al. [67] showed that in children born preterm at very LBW, the skin prick test overall and to mugwort and cat but not birch, timothy, dog, and house dust mite and IgE overall and to cat but not birch and timothy were significantly less positive than in full-term infants at 18 to 27 years of age. Asthma and low gestational age, but not atopy, were significantly associated with lower lung function values. Furthermore, it has been shown that the positive skin prick test to inhalants was associated with persisting wheezing but not with lower lung function in children preterm born at 10 years of age [68]. Mitselou et al. [69] studied the frequency of IgE to inhalants in two population-based birth cohorts, BAMSE and STOPPA. He showed that subjects preterm-born compared to those full-term-born had a significantly lower frequency of IgE to inhalants at 16 years of age but not at 4, 8, and 24 years or overall in the BAMSE study and no difference at 9–14 years in the STOPPA cohort. A combined meta-analysis of the results of BAMSE and STOPPA cohorts found that preterm birth was inversely associated with inhalant sensitization. Varying from previous studies, Mai et al. [32] found that very LBW had no impact on sensitization defined as positive skin prick test results in 12-year-old children. Several explanations for reduced sensitization in preterm children may be offered. Early microbial exposure can drive a Th1 immune response instead of a Th2 response. Another possibility might be that a low number of allergens have been investigated [70]. Finally, an immature immune system might not be able to develop a Th2 response. Accordingly, a longer gestation might induce a Th2 over Th1 response with allergic sensitization [71].

### 5.2. Infections

#### 5.2.1. Viral Respiratory Infections

Children born preterm have an increased rate of airway infections from infancy up to school age, but this susceptibility did not continue in adolescents [72,73]. Infants admitted to the hospital for respiratory infections in the first year of life were at increased risk of asthma. Stratification of the risk of asthma for gestational age showed a significantly higher asthma risk in preterm subjects, especially in those born <28 wGA. This risk persisted until after age 10 but was less pronounced after age 16 [74].

#### 5.2.2. Bronchiolitis and Asthma Development

Bronchiolitis among infants is associated with an increased risk of asthma. Bronchiolitis can be induced by several viruses. Specific organisms are considered important for the development of asthma. The most common infective pathogen is RSV in infants <2 years of age. There is evidence that bronchiolitis caused by RSV is not associated with asthma development both in term and preterm infants. Even if RSV bronchiolitis may lead to a 3.8-fold increased risk of recurrent wheeze and asthma, this association weakened over time, and it was no longer significant by the age of 13 years [75,76,77,78]. Most studies have shown that RSV bronchiolitis was not associated with atopy [75,79,80]. Preterm infants showed comparable figures [81,82]. The causal relationship between severe RSV infection and asthma in preterm infants was analyzed through prospective studies on palivizumab, an anti-RSV monoclonal antibody for preventing RSV infections in infants at risk [83]. Infants aged 1–3 years who received palivizumab experienced a reduction in wheezing days, reported recurrent wheeze, and the use of bronchodilators [84,85,86,87,88], but there was no major effect on lung function or the prevalence of physician-diagnosed asthma at 6 years of age [89,90,91]. Human Rhino Virus (RV) is another pathogen that is often associated with bronchiolitis. Those with bronchiolitis due to RV was four-fold more likely to develop recurrent wheeze than those with RSV bronchiolitis [92]. Moreover, in young children, low respiratory tract infections associated with RV compared to those associated with RSV were associated with more general practitioner attendances, more respiratory-related outpatient attendance, and more wheeze at follow-up [93]. In infants with viral wheezing, RV infections compared to RSV infections are the most significant predictors of asthma at the age of 6 years [94]. At 11 months of age, the risk factors for current atopic asthma at the age of 8 years were allergic sensitization, atopic eczema, and RV wheezing [95]. Atopy was a risk factor not only for asthma in RV bronchiolitis [96,97]. Premature birth is a risk factor not only for RSV disease but also for severe RV disease [98,99]. There are some data showing that in very preterm infants, bronchiolitis is more commonly caused by RV than by RSV [100]. However, studies on the development of asthma following early RV infection in children born preterm are missing. In preterm infants, infections favored a lower production of IFN-α and IFN-γ with a consequent impaired viral clearance [101], a T2 response with increased levels of IL-4 and IL-13 [101,102], eosinophil activation and chemotaxis, and higher levels of IL-17A [103].

#### 5.2.3. Chorioamnionitis

Chorioamnionitis is another independent risk factor for recurrent wheeze and asthma in preterm infants [104,105,106] with the highest risk in the very preterm ones and in African Americans [104,105]. Chorioamnionitis was not associated with pulmonary function impairment [107]. Moreover, in term infants, chorioamnionitis had no effect on the rate of wheeze at 3.5 years and 7 years [108], suggesting that the preterm lung is more susceptible to disruption by inflammation.

## 6. Breastfeeding

In preterm infants, reduced breastfeeding has been observed [109]. Various studies have found that breastfeeding is a protective factor against the development of recurrent wheeze and asthma. Exclusive breastfeeding during the first 3 months of life was associated with 30% reduced odds of childhood asthma [110]. Breastfeeding for at least 6 months was associated with a 24% reduction in the rate of “recent asthma”, with a 19% reduction in “recent wheeze”, and there was a strong inverse association between breastfeeding and asthma or wheeze up to age 2 [111]. Ever breastfeeding was associated with a 12% reduced risk of asthma in children aged 5–18 [112]. Human milk is a complex mixture with bioactive compounds that include cytokines, immune cells, proteins, oligosaccharides, microbiota, bacterial metabolites (short-chain fatty acids), and mRNA modifications that may prevent prematurity-associated asthma. More studies are needed to elucidate immunological and epigenetic mechanisms involved in asthma prevention [113].

## 7. Microbioma

Consistent with the hygiene hypothesis, missing microbial exposure in early life leads to immune system development with the onset of allergic diseases [114,115]. Accordingly, a reduced risk of asthma has been shown when there is a highly differentiated gut microbiota in early life [116]. Atopy and the development of asthma at 3 months of life are reduced by a high frequency of *Candida* and *Rhodotorula* in the composition of the microbiota profile through a dysfunction in the CD4 T-cell; in contrast, it is promoted by an abundance of *Bifodobacteria*, *Akkermansia*, and *Faecalibacterium* [117]. Furthermore, in the infant gut, the abundance of strains with anti-inflammatory properties (such as *Bacteroides*, *Roseburia*, and *Coprococcus*) may protect from asthma onset [118]. In preterm infants, microbiota colonization in the airways is enriched for RSV/HRV-A, *Haemophilus influenzae*, *Streptococcus pneumoniae,* and *Moraxella catarrhalis,* which are associated with persistent wheeze and asthma [119] at 5 years [120] as well as with the later development of pneumonia or bronchiolitis [121]. In preterm infants, increased asthma development has been linked to a reduced abundance of *Bacteroides*, *Bifidobacterium*, *Lachnospira*, *Veillonella*, *Faecalibacterium*, and *Akkermansia* and a greater abundance of *Clostridium difficile, Clostridium neonatale*, *Staphylococcus*, *Proteobacteria*, and *Enterobacteriaceae* (*Klebsiella*, *Enterococcus*, and *Escherichia*) in the gut and even in the airway microbiome [122]. This complex led to enhanced eosinophilic and neutrophilic recruitment in the lung, increased IgE levels, bronchial hyper-responsiveness, and decreased IL-10 production and Treg function, which may contribute to asthma occurrence [122,123]. Interestingly, a lower abundance of *Proteobacteria*, *Enterococcus*, *Lactobacillus,* and *Acinetobacter* and the dominance of *Bacteroides* and *Bifidobacterium* in the intestinal microbiota during the first month of life was associated with the development of allergic sensitization [122]. Several factors such as cesarean delivery and the use of antibiotics can explain the different composition and poor diversity of the microbiome in preterm infants compared to term infants. Nutrition can also play a role. Breastfeeding is associated with a dominance of *Bifidobacteria* in the gut, which is a marker of healthy microbiota development. Gut dysbiosis may negatively affect the immune response [124], predisposing infants to increased susceptibility to infections and asthma development [125,126].

### 7.1. Delivery

The mode of delivery represents a risk factor for asthma. An increased risk of childhood asthma in children born through cesarean section has been found in several systematic reviews [127,128]. An increased asthma hospitalization risk only among cesarian-delivered preterms has also been shown [129]. There are differences between delivery modes at birth that progressively diminish over the first year of age. Mother-to-infant transmission was compromised in vaginally delivered infants who shared only 72% of gut microbes with their mother, while cesarean-delivered infants shared 41%, with less sharing of *Bifidobacterium* and *Bacteroides* (specifically *Prevotella*), while that of *Enterobacteriaceae* was maintained [130]. In cesarean-delivered infants, the gut microbiota consists of germs of the maternal skin, mouth, and surrounding environment. There was an abundance of *Propionibacterium*, *Corynebacterium*, *Haemophilus parainfluenzae*/*influenzae,* and *Staphylococcus* spp. and also of *Clostridium perfringens*, *Veillonella dispar*/*V. parvula,* and *Enterobacteriaceae* (*Enterobacter cloacae* and *oxytoca*, *Klebsiella* species, *Enterococcus*), which significantly differed from the gut microbiota of vaginally delivered infants. They displayed maternal vaginal and gut microbes enriched in *Bifidobacterium*, *Bacteroides* (*Prevotella*), *Parabacteroides*, *Enterobacteriaceae* (*Escherichia*), *Sneathia*, and *Lactobacillus* spp. [130].

### 7.2. Antibiotics

Preterm infants are more frequently exposed to antibiotic therapy [131]. A recent meta-analysis demonstrates that antibiotic treatment during pregnancy is associated with an increased risk of asthma during childhood [132]. A revision of 52 articles [133] showed that exposure to antibiotics was significantly associated with increased odds of childhood asthma in infants. The risk of asthma is increased after postnatal antibiotics but not after prenatal use [134,135,136]. Patrick et al. [137] found that antibiotic use in the first year of life was associated with a two-fold increased risk of asthma at age 5 years. The reduction in the incidence of asthma from 2000 to 2014 was associated with decreasing antibiotic use in infancy. Morata et al. [18] found that antibiotic use in the first 3 years of life was a risk factor for asthma at school age in children born preterm. Antibiotics may act by disrupting gut microbiota, which can predispose children to asthma development by altering the immune response. Maternal antibiotic exposure before or during labor is associated with reduced *Bacteroides* and *Parabacteroides* and increased *Clostridium* and *Enterococcus* at 3 months of age [138]. Along this line, both asthma and antibiotic exposure are associated with a loss of microbial diversity, a reduction in *Bacteroidetes* (*Rikenellacea*), *Faecalibacterium*, *Roseburia*, and *Ruminococcus bromii*, and an increase in *Clostridium perfringens* [137,139]. The increased risk of asthma does not seem to depend on respiratory infections that require the use of antibiotics; in fact, the results were confirmed even by excluding patients who presented respiratory infections [136].

## 8. Tobacco Smoke

Prenatal exposure to tobacco smoke increased preterm delivery and the risk of asthma later in life independently from postnatal exposure [29,140,141,142,143,144]. It is unclear whether maternal exposure to secondhand smoke during pregnancy can affect the prevalence of preterm birth [145,146]. The increased risk for asthma is caused by both maternal smoking [147] and exposure to environmental tobacco smoke during pregnancy [148,149]. Nicotine crosses the placental barrier and accumulates in the fetus with serum concentrations higher by 15% than maternal ones [150]. Nicotine induces placental vasoconstriction, which leads to reduced oxygen and nutrient contribution. This impacts alveolar development, breathing actions, the growth and maturation of airways and lungs, and the subsequent development of respiratory diseases [151,152]. Maternal smoking impaired lung development with increasing risk of BPD in preterm [153,154,155]. Regarding mechanisms of asthma onset, nicotine impaired cellular differentiation and airway development, which leads to reduced respiratory function in infants born preterm of smoking mothers compared to those of nonsmokers [156]. Macaubas et al. [157] showed that maternal smoking was associated with an increased risk of asthma and atopy at 6 years of age and with a Th2/Th1 unbalance due to lower levels of serum interleukin 4 and interferon-gamma in cord blood. Furthermore, in mice, maternal smoking during pregnancy induces airway remodeling with hyperplasia of the airway smooth muscle, collagen III deposition, mites-induced mast cell numbers and methacholine responsiveness in house-dust-mite-exposed offspring [158], and significantly exacerbated HDM-induced airway eosinophilic inflammation with increased reactivity, mucus secretion, and T2 cytokine levels in the offspring [159]. Exposure to maternal smoke increases the risk of lower respiratory tract infections in infants, which may trigger asthma. Several studies have shown a transgenerational inheritance of smoking effects [160]. Finally, epigenetic regulations due to the environment pass to the offspring. Prenatal maternal smoking and rising asthma symptoms such as wheezing at age 10 were associated with AXL methylation at birth, measured in bloodspots, in two population study cohorts [161]. Exposure to prenatal maternal tobacco smoke was associated with higher methylation levels in the AHHR gene measured in whole blood and asthma in 8- to 21-year-old Latino children [162]. Smoking cessation before or in the first trimester of pregnancy was associated with a reduction in preterm delivery [163,164,165,166,167,168,169]. This should push more efforts for the timely cessation of smoking in women.

## 9. Family History

Children with parental asthma have an increased risk of asthma than children without parental asthma, which is 3-fold greater for maternal asthma and 2.4-fold greater for paternal asthma [170]. Familiarity with asthma represents a risk factor for the development of asthma even in preterm infants, as demonstrated by a long-term observational study evaluating preterm infants <32 weeks or weighing <1500 g [171]. Genetic factors certainly favor the development of asthma in children of asthmatic parents, but there is also some evidence that women with asthma have an increased risk of preterm labor [172].

## 10. Prevention Strategies

Preventive strategies to reduce the risk of asthma are numerous and still being studied. Among interventions for preventing asthma in children born preterm, the administration of daily probiotics seems to have no effect on asthma diagnosis [173]. Other strategies have been studied in full-term babies with mixed results [174,175]. The transplacental passage of vitamin D occurs above all in the third trimester, so preterm infants are often deficient [176], but its supplementation during pregnancy in case of parental atopy had no effect on the development of asthma in the offspring [177]. A systematic review and meta-analysis suggests that bacterial lysates decrease the risk of wheezing episodes and asthma exacerbations [178]. However, further studies in preterm infants that can clarify the actual usefulness of preventive strategies are needed.

## 11. Conclusions

Many factors contribute to the increased risk of asthma in preterm infants. Intrinsic factors such as structural abnormalities (smaller airways), immune response to viral pathogens or aeroallergens, inflammation, and microbiome diversity play a pathogenetic role. Several risk factors including delivery, pollution exposure, feeding, and drugs influence the development of asthma. These findings may indicate that asthma in preterm babies may be associated with a unique pathway that may be different from those in full-term infants. However, it remains difficult to distinguish the true relationship between causes and effects. This hampers the implementation of practical strategies for mitigating the onset of asthma. Further studies in preterm infants for clarifying asthma endotypes and subgroups at risk of asthma and developing successful prevention strategies are warranted.

## Figures and Tables

**Table 1:** Risk factors associated with the development of childhood asthma in preterm babies vs. full-term babies without the risk factors (if not otherwise specified).

Risk Factor	Risk of Asthma (Odds Ratio/Relative Risk and 95% Confidence Intervals) *
Very preterm (<32 wGA)	3.9 (3.2–4.8)
Moderate–late preterm (33–36 wGA)	1.7 (1.4–2.0)
Extremely low birth weight (<1000 g)	1.8 (1.1–1.4)
Very low birth weight (<1500 g)	1.43 (1.34–1.54) (compared to LBW)
Childhood weight gain >700 g/month	4.47 (2.58–7.76)
Oxygen supplementation in VLBW	4.3 (1.3–14.0)
Respiratory infections in <28 wGA	2.2 (1.59–3.09) (compared to <28 wGA without respiratory infections)
RSV bronchiolitis	3.8 (3.23–4.58) (no effects of palivizumab)
RV wheezing	25.6 (8.2–79.6)
Chorioamnionitis in preterms	2.9 (2.6–3.3) (compared to chorioamnionitis in full-terms)
Airway bacterial colonization	4.57 (2.18–9.57)
Delivery by caesarian section	1.2 (1.04–1.39)
Early antibiotic exposure	2.2 (1.2–4.2)
Maternal smoking in pregnancy	1.35 (1.13–1.62)

* According to the data cited in the paper. VLBW, very low birth weught; RSV, respiratory syncytial virus; RV, rhinovirus.

## Data Availability

https://pubmed.ncbi.nlm.nih.gov.
